# The Translation of Policy to Person: A Qualitative Analysis of Elite Athletes’ Perceptions of Pregnancy in the United Kingdom

**DOI:** 10.1007/s40279-025-02191-9

**Published:** 2025-03-16

**Authors:** Catherine V. Caro, Storm Trow, Zoë Bell, Angela C. Flynn, Fiona Lavelle

**Affiliations:** 1 Department of Nutritional Sciences, School of Life Course and Population Sciences, King’s College London, 150 Stamford Street, London, SE1 9NH UK; 2 School of Population Health, Royal College of Surgeons in Ireland, Dublin, Ireland

## Abstract

**Background:**

An increasing number of female athletes are navigating an athletic career alongside pregnancy. Limited qualitative research has investigated the experiences of elite athletes in the United Kingdom (UK). This study aimed to explore the experiences of elite athletes in the UK as they navigated pregnancy, employing a socio-ecological framework to inform future research and policy recommendations on sport participation during pregnancy.

**Methods:**

A descriptive qualitative study design, adopting a relativist ontology and constructivist epistemology, was implemented. Semi-structured online interviews were conducted with elite athletes ≥ 18 years old, who resided in the UK, and who trained and/or competed at the highest level of their sport prior to and/or during pregnancy. Interviews were recorded, transcribed and analysed using reflexive thematic analysis.

**Results:**

Eleven athletes (mean age 31 ± 3 years) from nine team and individual sports participated in the study. Four key themes were developed: (1) *From the Podium to Parenthood: Institutional versus Individual Influence on Reproductive Planning*; (2) *Is My Career Over? Micro Level Support versus Macro Level Doubt and Worry*; (3) *Athlete to Mother: Internal Conflict to Community Role Model*; (4) *Navigating the Bump: Individual Drive to Tackle Systemic Gaps*.

**Conclusions:**

Findings highlight the complexity female athletes face when navigating pregnancy, motherhood and elite sport. There is a need for high-quality research focusing on preconception and pregnancy-specific training and nutrition modifications for elite athletes, particularly regarding nutrient intake and supplementation. Additionally, efforts to improve the translation of evidence-based research into practical applications remain essential.

**Supplementary Information:**

The online version contains supplementary material available at 10.1007/s40279-025-02191-9.

## Key Points

**Table:** 

Despite new pregnancy and maternity guidelines, elite athletes still face challenges in navigating unclear policies, highlighting a need for more tailored support.
Elite athletes experience a significant gap in pregnancy-specific nutritional guidance, often receiving generic advice that fails to meet their unique nutritional and performance needs during pregnancy.
Balancing athletic and maternal identities remains challenging for elite athletes, emphasising the need for stronger support networks and open discussions about preconception, pregnancy, and motherhood in sport.

## Introduction

Female athletes train and compete at an elite level during their reproductive years and an increasing number of athletes reach the peak of their career during this time [1, 2]. Over the last decade, female athletes have been reluctant to postpone having children until after their athletic careers. They have begun to push the boundaries of sociocultural expectations regarding training and competing in elite sport during pregnancy [3, 4]. However, research and evidence-based policies to understand and support an elite athletic career alongside pregnancy and motherhood remain in their infancy [3–6].

Pregnancy is associated with significant physical, mental, and emotional health changes [7–9]. The role of exercise and nutrition during pregnancy is well documented in its short- and long-term impact on pregnancy, maternal and childhood outcomes [8, 10–13]. However, this evidence and further recommendations are predominantly limited to the general population and not tailored for elite athletes [14–17].

Recently, international and national organisations, governing bodies, and research groups have begun to conduct research and create policies to better support this often-overlooked group of athletes [18–23]. Contemporary evidence, including two systematic reviews and meta-analyses, suggests that elite-level training across all sports does not lead to adverse maternal and foetal health outcomes and in most cases, pregnant athletes can continue training [6, 24–30]. However, historically there have been safety considerations and theoretical concerns associated with exercise during pregnancy, specifically long-duration and high-intensity cardiovascular or strength training [21]. There is a paucity of high-quality evidence summarising appropriate training modifications for pregnant elite athletes and evaluating the potential impact on performance [21, 31].

The lack of reliable evidence and tailored recommendations for elite athletes, coupled with the unique demands of each athlete's sport, individual perinatal experiences, and prevailing socio-cultural narratives surrounding motherhood, sport and performance, creates significant practical challenges for elite athletics navigating pregnancy [2, 5, 21]. Recent literature, primarily from North America, Australia, Europe and the United Kingdom (UK), exploring the experiences of elite female athletes during pregnancy highlights that athletes are often unsatisfied with the recommended modifications or advice for their training and exercise because of safety concerns and uncertainty [2, 4, 6, 28]. Uncertainty can stem from medical professionals regarding the advice they provide, from athletes questioning the guidance given, or from insufficient supporting evidence for the advice itself [2, 4, 6, 28]. Further research suggests that there is a lack of evidence-based recommendations, although a body of evidence is growing [6, 32, 33].

A socio-ecological framework (SEF) is a theoretical model used to understand the multiple levels (intrapersonal, interpersonal, community, organisation and policy) of influence on individual behaviour, recognising that these influences span from personal to broader institutional and societal factors [32, 33]. When considering a SEF to explore the experiences of athletes during pregnancy, the majority of research is conducted at the 'micro' level, intrapersonal, interpersonal and, increasingly, community. This research has led to evidence-based recommendations and actionable steps to create policy-level support for pregnant athletes [2, 34, 35]. However, despite this, most international federations still lack policies or guidelines providing sport-specific support to female athletes during or after pregnancy [36]. Data from professional female footballers in England advocate for this support, highlighting the incompatibility of contracts, maternity policy and motherhood [37]. The UK has responded to various calls for better support for elite athletes during pregnancy [38, 39]. A qualitative study was conducted to inform a set of first-of-its-kind pregnancy guidelines [39–41]. While most participants included had not experienced pregnancy, the research provided athletes and governing bodies with preliminary information on available health resources, frameworks and funding protection that had previously never existed within the UK or internationally. Limited research has been conducted to investigate how these policy-level influences are affecting athletes after implementation.

While the highlighted qualitative evidence demonstrates progress within the UK and internationally, sex- and gender-specific research, education and policy require sustained effort and commitment [42]. As the number of female athletes participating in sport at an elite level increases, it is essential to continuously understand their experiences and perceptions of pregnancy and career development, sex-specific physical, mental and nutritional requirements and contractual conditions of females in elite sport [37]. This is particularly relevant in progressive environments like the UK where evidence-based guidance and policies are available.

Capturing elite female athletes’ perspectives is critical for sports and exercise science research and subsequent policy development and implementation—“nothing about us, without us” [43]. This study aimed to explore the experiences of elite athletes in the UK as they navigated pregnancy, employing a SEF to inform future research and policy recommendations on sport participation during pregnancy.

## Methods

The study is reported in line with the consolidated criteria for reporting qualitative research (COREQ) guidelines (see Online Resource 1 in the electronic supplementary material [ESM]). To enhance transparency, the protocol was registered with Open Science Framework Registries [44]. This study was approved by King’s College London Research Ethics Board (Ref No: LRS/DP-22/23-34763).

### Study Design and Procedure

A descriptive qualitative study design was implemented [45], as researchers wanted to describe the experiences of elite athletes during pregnancy in relation to their training and support during this important life stage and explore their perceptions of current evidence and policies [46, 47]. A relativist ontology was adopted in the understanding that reality is not singular and is based on an individual’s past experiences and the context. A constructivist epistemology was undertaken given the subjective nature of knowledge, recognising that individuals actively construct meaning based on their own experiences, beliefs and social contexts [48]. Further, an interpretivist approach was used acknowledging that knowledge is subjective and that it is created through a lens of participant interpretation. Semi-structured interviews were conducted to allow for in-depth discussion and individual reflection and interpretation of experiences, without the influence of other athletes [46, 47].

Between March and June 2023, elite athletes were recruited through social media platforms (e.g. Twitter, Instagram, Facebook and LinkedIn), direct contact (e.g. email and King’s Sport) and word of mouth via a purposeful and snowball sampling approach [49]. Eligibility criteria included athletes aged 18 years or older, resident in the UK, who have trained and/or competed at the highest level of their sport immediately prior to and/or during pregnancy within the last 10 years. Athletes’ training and performance calibre was defined using the 6-tiered Participant Classification Framework; Tier 4: Elite/International Level or Tier 5: World Class athletes were included in this study [31]. Prior to participation, athletes were electronically provided with the study information sheet, consent form and brief questionnaire. This questionnaire included items such as date and place of birth, ethnicity, type of sport played at an elite level, highest level of training or competition achieved, number of children, and if they had retired from sport. No relationship was established prior to study commencement. A minimum of 24 h was provided between participants receiving this information, and completed documentation and consent being obtained.

Participants engaged in one 45-min one-on-one semi-structured interview via Microsoft Teams with one of two female researchers completing their MSc in Nutrition (CC, ST). The researchers introduced themselves and provided the participants with a brief introduction to the study. Participants were informed that all opinions were valid, confidentiality would be maintained and they could withdraw at any point. The interview topic guide was informed by sport, pregnancy, and postpartum-related literature and piloted on two US-based elite athlete mothers [2, 40, 41, 50]. The topic guide related to athletes’ experiences in elite sport, specifically during pregnancy and the postpartum period (see Online Resource 2 in the ESM for topic guide). The postpartum period was defined as the period from immediately after delivery until the infant is 12 months of age [51]. All interviews were audio-recorded, transcribed verbatim by Microsoft Teams, and checked for accuracy and precision by the researchers (CC, ST). Field notes were made after each interview to manage researcher’s reflections on their positionality. This reflection acknowledges that qualitative researchers are shaped by their own perspectives and biases within the research context [52]. All authors are female with research interest in women’s and maternal health. Two authors have experience competing at an elite sporting level and one author has experienced pregnancy. Transcripts were not returned to participants for comment, correction or feedback.

### Data Analysis

Pre-interview survey data were summarised using SPSS Statistics (IBM Corporation, Armonk, NY, USA, 2013). Transcripts were imported into NVivo (QSR International Pty Ltd, Doncaster, Victoria, Australia) for thematic analysis. Reflexive thematic analysis was chosen for this study in order to examine each participants’ perspectives, highlighting similarities and differences and uncovering expected and unexpected insights [53]. This research was part of a wider study encompassing both pregnancy and postpartum. All instances relating to the pregnancy period were included in this study. The sample was assessed as having sufficient information power, given the focused aim and tight sample specificity of the study, and the experience of the moderator and wider research team to generate focused and rich data [54]. The researchers (CC, ST) listened to the audio recordings and reviewed and revised the automated transcripts. To familiarise and fully immerse themselves in the data prior to formal analysis, the researchers (CC, ST) read and reread the transcripts, and shared summaries of each interview [55, 56]. All transcripts were initially coded semantically (CC) to recognise the concepts directly communicated by participants. Codes were grouped together to generate potential themes, inspected for overlap and refined as needed by CC. Themes were then defined, named and described. Each theme represented a pattern of shared meaning underpinned by a central organising concept rather than summarising the data [53]. Four researchers (CC, ST, FL, ZB) reviewed and challenged each theme through critical dialogue [57]. Further interpretation of the themes beyond their surface level and how they align with a SEF was conducted by categorising the data from each theme into (1) intrapersonal, (2) interpersonal, (3) community and organizational and (4) policy. A SEF assumes that interactions between individuals and their environment are reciprocal, implying that an individual is influenced by one's environment and the environment is influenced by the individual. This interpretation is identified in the results section and contextualised in the discussion. Direct quotes were extracted from the data to illustrate the themes. Identifiers, such as name and name of sport, were removed from the quotes and replaced with pseudonyms and type of sport, respectively.

## Results

Fifteen athletes responded with interest in participating in the study. Two athletes were deemed ineligible due to either athlete calibre or country of residence, and two declined to participate after initial interest. Each interview lasted between 23 and 64 min. Eleven athletes (mean age 31 ± 3 years) from nine team and individual sports participated in this study (see Table 1). Nine of the athletes had between one to three children and were not pregnant at the time of the interview. Two of the athletes were in their final trimester of their first pregnancy, between 38 and 39 weeks’ gestation, and training at a modified level. Immediately prior to pregnancy and during the first trimester, both trained and/or competed at the elite level.

**Table 1:** Demographic, pregnancy and sporting participant characteristics

Participant characteristics (*N* = 11)	
Duration of elite sport participation [years], mean (SD)	10 (5)
Years since most recent pregnancy [years], mean (SD)	2 (1.5)
Retired, *n* (%)	0 (0)
Number of children, *n* (%)	
Currently pregnant	2 (18)
1	8 (73)
2	0 (0)
3	1 (9)
Type of sport, *n* (%)	
Team	5 (45)
Sprint and endurance	2
Ballgame	3
Individual	6 (55)
Sprint and endurance	3
Power	1
Precision and technical	1
Combat	1
Highest level of competition, *n* (%)	
Olympic Games or World Championships (Tier 5)	8 (73)
Paralympic Games or World Championships (Tier 5)	1 (8)
Elite/International competition (Tier 4)	2 (18)
Ethnicity, *n* (%)	
White British	8 (73)
White Other	1 (9)
Black Caribbean	2 (18)

Four key themes were generated: (1) *From the Podium to Parenthood: Institutional versus Individual Influence on Reproductive Planning*; (2) *Is My Career Over? Micro Level Support versus Macro Level Doubt and Worry*; (3) *Athlete to Mother: Internal Conflict to Community Role Model*; (4) *Navigating the Bump: Individual Drive to Tackle Systemic Gaps* (Theme flowchart available in Online Resource 3, see ESM). Themes are mapped to the SEF in Table 2.

**Table 2:** Socio-ecological framework mapping of themes

Socio-ecological framework		Theme			
Examples of influences	Levels of influence	(1) From Podium to Parenthood: Institutional versus Individual Influence on Reproductive Planning	(2) Is My Career Over? Micro Level Support versus Macro Level Doubt and Worry	(3) Athlete to Mother: Internal Conflict to Community Role Model	(4) Navigating the Bump: Individual Drive to Tackle Systemic Gaps
**MACRO**					
Policies, regulatory agencies, health system, political/geographical environment	Policy	Guidance or policy protecting funding			Regulation on supplements
(National and sport) governing bodies, public agencies, sponsors, sport leagues and competitions	Organisational	Competition timing	Unable to disclose to support; staff ill-equipped		Lack of support/knowledge/evidence—their dedication not reciprocated; lack of direction; unclear training adaption; no specific nutrition guidance
Community resources, social and health services	Community				Follow NHS advice; no specific nutrition guidance
**MICRO**					
Social networks, teammates, coaches, families, peers	Interpersonal		Familial and peer support	Future support; aspirations	Growing community of athletes
Knowledge, skills, attitudes, values, preferences, emotions, behaviours	Intrapersonal	Injury; fertility and hormonal	Uncertainty	Emotions; guilt	Internal fight to stay in the system; waiting

### From the Podium to Parenthood: Institutional Versus Individual Influence on Reproductive Planning

Participants emphasised that their respective governing bodies and upper management are instrumental in providing top-down policies and guidelines to support preconception and pregnancy planning. While participants acknowledged that significant progress is still required, they praised The Female Health and Performance Team and UK Sport for assembling female-specific multi-disciplinary teams and resources, and for facilitating the 2021 Pregnancy Guidance. Participants described how this guidance provided information on key areas of concern such as funding and/or sponsorships during pregnancy, acting as a protective factor for career security.

Without the maternity policy, like you can't fund it and or certainly you wouldn't want to risk having your funding cut and losing literally your entire income whilst being pregnant … So, I think that's been like quite a big game changer. – Mary, team endurance sport

Participants explained how becoming pregnant or delaying pregnancy is reliant on timing around key competitions, such as the Olympics, or during the rehabilitation of long-term injuries. Participants described conversations with their partners about the ‘window of opportunity’ during an injury or break from competition, which might be their only chance to actively plan a pregnancy without the need to delay for upcoming competitions.

I very much was focused on trying to go to make Olympics, so kids or getting pregnant was never in my head before that point…Then the Olympics got delayed by year, which then delayed my pregnancy plans. – Lily, team ball sport

Athletes also described their concerns around their ability to conceive due to menstrual cycle irregularities and hormonal imbalances. This was often highlighted by medical professionals. In some instances, participants shared that despite their efforts to plan pregnancy, it did not always go as planned, similar to the general population. For example, pregnancies occurring unexpectedly, often before or during key competitions, potentially derailing key performance targets, or taking longer than expected due to known or unknown fertility and hormonal issues.

[name] wasn’t planned actually … I've been told [by team medical professionals] due to [sport] I was actually infertile and I had polycystic ovaries and that was kind of the form of contraception, but that clearly wasn’t the case. – Mary, team endurance sport

### Is My Career Over? Micro Level Support Versus Macro Level Doubt and Worry

Upon becoming pregnant, many participants described feeling uncertain. They tried to understand what currently accessible resources would be beneficial that would help navigate these changes, and what would be next for their career.

I went and done the test then it said pregnant, and I was like oh no that's my career over with. – Anna, individual power sport

Participants expressed their reluctance to disclose their pregnancy to coaches, support staff, and teammates due to a fear of appearing not committed or being cut from the team and risking their financial position. This led to some participants pretending to be injured, to differing degrees of believability or strategically disclosing the information where ethically it would be protected.

I [only] told the team doctor and the team psychologist ... and we kind of had a management strategy … the main reason I wanted to speak to those two about it is because I knew that their jobs were, that they had to keep patient confidentiality. – Isabelle, individual endurance sport

Athletes reported turning to their sport’s medical professionals to guide their understanding of what adaptions were required during pregnancy, including immediate training and nutrition modifications. While safety for mother and baby was always emphasised as the number one priority, athletes felt medical professionals were ill-equipped to advise on further modifications due to the limited research in their population. This lack of evidence, educational resources and guidance created a mental strain on some athletes. Athletes reported extreme fear about the impact of training on the success of their pregnancy; a burden which resulted in these athletes significantly reducing their training during pregnancy.

We [athletes and team medical professionals] looked for research or anything like that. It's very, very minimal. – Grace, individual technical sport

Participants expressed gratitude towards those who provided immediate support upon becoming pregnant, those potentially considered in the more micro levels of influence. This included family and personal relations, coaches who were described as having experience working with pregnant athletes, teammates and female health specialists. These individuals provided the athletes with reassurance of their return to sport at the same level when the athletes faced insecurities.

If I didn't have an understanding partner, there's no way I would have been able to do what I do. – Grace, individual technical sport

### Athlete to Mother: Internal Conflict to Community Role Model

Participants discussed the significant decisions, challenges, and emotions faced as they navigated their newfound dual identity status as an athlete and mother. They expressed ideas of loss of control, guilt and potential sacrifice between their personal goals and dreams of being a professional athlete and becoming a mother. Participants described feelings of uncertainty as they tried to understand what would be next for their career, emphasising that being an athlete and mother often felt mutually exclusive. Support from macro-level influences such as their sporting bodies and relevant health professionals was identified as a barrier or enabler in managing the identity split and potential career diversion or delay.

I struggled with that, with the pregnancy because I had no control, what was happening with my body. I just felt like it was like a progressive injury for nine months... I think just a psychologist in general is important because there is a change of identity. – Grace, individual technical sport

Throughout the pregnancy journey, participants hoped their experiences would positively inform any future athlete deciding to become pregnant. They expressed desires to pave the way for others, to highlight that it can be done and to act as role models.

Part of me coming back is I do want things to be different for the next woman who comes and does this. – Charlotte, team endurance sport

### Navigating the Bump: Individual Drive to Tackle Systemic Gaps

Participants described the guidance they received in terms of training and nutrition support while pregnant. In many instances, the expectations, aspirations, and guidance on what the athletes could and/or should do did not align, as they wanted more robust direction and evidence. Participants emphasised the need for more research into the physiological effects of pregnancy within the elite athlete population. They reported that their dedication to sport was not reflected in the support they received.

More could be done. I do think it's frustrating because I feel like you give so much to the sport and when you don't feel every stone's turned to help you, it can be quite frustrating, especially when I feel like there must be simple answers out there. – Charlotte, team endurance sport

Participants described the gender/sex gap in sport and research, acknowledging that pregnancy is specifically a burden to one sex, the one that has historically been neglected in this area, and it makes sense there is limited evidence. However, participants felt optimistic about the future.

Men don't have this problem. So, the research behind it won't be there historically. So as the game grows, hopefully the research will grow with it. – Emma, team ball sport

Participants emphasised the importance of flexibility and individualisation of training programs due to diverse pregnancy experiences. However, many described uncertainties in adapting their training programs for pregnancy. They further highlighted concerns in the maintenance of cardiorespiratory and muscular capacity and performance level with the long-term plan of returning to sport postpartum. They struggled with the lack of direction from coaches and governing bodies in how to stay involved in sport during their pregnancy. Participants stressed that to navigate pregnancy effectively, it was important to have clear goals and expectations from meetings with coaches and the management team, to ensure going beyond a ‘tick box’ activity. They expressed feelings of dismissal, and feeling as though they were not being taken seriously when discussing a return to sport. This lack of structured support often led to negative feelings amongst the athletes as they grappled with the change of no longer being involved in the elite support system. Further, athletes were often told to follow generic advice from the NHS including the cessation of all long-duration or high-intensity exercises, “well you can’t do that now” and to follow a list of what one cannot eat rather than what one can and should eat.

Gaps in education and resources varied across participants and sports. When participants felt most supported with their training and overall goals during pregnancy, they described having access to appropriate health and sporting professionals and female health specialists, such as coaches, nutritionists and physiotherapists. Additionally, proactive coaches referenced the latest evidence-based research in pregnant elite athletes to guide training management strategies. They implemented target heart rate zones and strength exercises modified to accommodate the physiological changes of pregnancy, amongst other supportive strategies. Furthermore, key support systems to athlete’s mental and physical health and wellbeing during pregnancy were at micro levels, growing a community of athletes competing at elite levels during pregnancy. This community was reinforced through a ‘WhatsApp’ group to facilitate communication. This group facilitated a space for athletes to share personal accounts of their own management strategies, providing peer support and alleviating some of the barriers faced by athletes training during pregnancy. This group also benefits athletes in providing role models for peers to instil hope of returning to sport in postpartum.

One of the largest and consistently highlighted gaps athletes described in their support was around nutritional advice. Participants described a lack of tailored advice for athletes, “I got nothing at all,” and often felt that there was a lack of educational resources and evidence-based guidance. Reflecting the superimposing of sport guidance seen generally for female athletes, again athletes described how pregnancy recommendations were not specific to them, rather based off the general population. They further highlighted contrasting Sports Nutrition principles for their current pregnancy status.

I talked through the doctor to make sure that I'd be safe to continue doing things like taking caffeine, which was originally part of my nutrition strategy, and I didn't know what food I was and wasn't allowed to eat. – Isabelle, individual endurance sport

Athletes also raised issues around lack of access to triple batch tested or “Informed Sport” dietary supplements that are necessary to take during pregnancy. Participants understood the importance of taking supplements such as folic acid, however they struggled to find a supplement solution that worked for them both as a mother and as an athlete.

A big one for me is what you can and can't take when you're pregnant…Whenever I ask those questions to the doctor, team nutritionist, they basically redirect them to midwife...the midwife advised on some supplements like folic acid and iron and other things to take. But one thing I have struggled with is a lot of the supplements that you are meant to take are not Informed Sport tested. So if antidoping has a problem with them then that's a problem. And you can't get around some of it. – Lily, team ball sport

## Discussion

These UK-based findings further illuminate the inherent complexities female athletes face when navigating pregnancy, motherhood and elite sport previously reported in wider qualitative research [4]. While the UK has begun to provide evidence-based pregnancy and maternity guidelines and policy, participants in this study continue to expand our understanding of these current research, guidelines and policies supporting elite athletes during pregnancy. Although this research was conducted before specific policy updates, such as the 2023 UK Sport Pregnancy Guidelines [58, 59], it highlights that overall gaps still remain. Strikingly, this research highlights a severe deficit in understanding of nutritional needs, appropriate modifications and evidence to support athletes’ nutrition during pregnancy. Furthermore, despite positive developments in supportive policy, there is limited knowledge translation of policy and evidence-based research for practical application and education in preconception and pregnancy specific to elite athlete training and nutrition. A SEF will be used to interpret these findings with existing literature and generate research-based recommendations.

### Policy

Policy level support was variable across sports and governing bodies. While various teams and organisations have begun to address the lack of support and uncertainty surrounding pregnancy and maternity guidance and policies for elite athletes [5, 38], the majority of governing bodies lack evidence-based, athlete-centred policies [5, 38]. UK Sport has pioneered the development of pregnancy and maternity guidelines and policy in elite sport and established a team of female health specialists and researchers to support and collaborate with elite athletes [39–41]. Most recently, UK Sport updated their pregnancy guidelines. The guidelines reflect more inclusive pregnancy and maternity funding policies, increase visibility of athletes who have successfully navigated pregnancy and elite sport and include more evidence-based recommendations to athletes, coaches, healthcare providers and sporting governing bodies [58, 59]. Additionally, England Rugby implemented new maternity leave policies for their players, and England Netball launched NETBALLHer, a program to better educate women and girls at all levels of sport about their bodies across different life stages [60, 61]. While these initiatives help break down barriers, this study highlights that gaps may persist for elite athletes in the UK. As these interviews were conducted before the 2023 UK Sport’s guideline update, it remains uncertain whether those changes will lead to meaningful improvements, suggesting that policy and resources may fail to translate down to athletes and their wider support who could implement some of the knowledge. Further research is warranted.

This research has shown that navigating a sporting career for athlete mothers is complex, as it is often tied to the constraint of fixed competition structures and timing, resistance of cultural norms such as the prioritisation of caregiving and self-sacrifice and limited structural support [4, 62]. Previous feminist poststructuralist discourse analysis draws attention to how the male-dominated sport system often overlooks or inadequately addresses female-specific needs [63, 64]. However, as athletes continue with sport careers during pregnancy and return to sport postpartum, the institutional acceptance and support of motherhood in sport should continue to shift to better support the needs of female athletes [63, 64]. While this current study used a SEF to clarify policy translation and influences across levels, future research should consider using a critical feminist lens to further explore how these policies, structures and infrastructures can impact female health.

Additionally, the findings raise a key consideration for regulatory bodies associated with the potential negative consequences of consuming the recommended pregnancy supplements for their sporting career. Participants struggled to find anti-doping approved folic acid supplements. Current UK guidance advises women to take 400 µg of folic acid daily before pregnancy and throughout the first 12 weeks to reduce the risk of neural tube defects [65]. The use of dietary supplements is common amongst elite athletes for management of micronutrient deficiencies and to enhance performance [66]. Although the phrase ‘food first’ is widely followed within sports nutrition and has been acknowledged to optimise performance, it may be ‘food first but not always food only’ [67]. This highlights the acceptance of supplementation to enhance performance beyond the capabilities of food. Antidoping codes exist to govern dietary supplementation intake in elite sport, however, non-performance supplements do not require the same standard of regulations [68]. While unlikely, inadvertent ingestion of banned substances due to cross-contamination is a necessary consideration of elite athletes taking any kind of supplements, such as folic acid and caffeine [66, 69]. Therefore, to support preconception and pregnancy health in this group, an approved folic acid supplement is required. This further supports the dichotomy participants expressed in a central message of this research, athlete versus mother; that elite sport participation and motherhood can feel mutually exclusive.

### Organisational and Community Findings

Organisational and community-related findings underscore the intricate interplay between elite sport participation and wider institutional support structures necessary to navigate this complex athlete-mother terrain effectively. Participants identified key factors that enabled or hindered their success navigating elite sport and pregnancy. These factors included heart rate monitoring, clear training plans and evidence-based sport-specific modifications and were aligned with actionable steps recommended by previous evidence [2, 23, 34]. Recent research shows no difference between elite athletes and physically active non-elite females and their ability to get pregnant, have healthy babies and return to sport. It also highlights that maternity leave is necessary and has a significant impact on postpartum performance [26]. Guidelines have addressed pregnancy and maternity protection concerns [70, 71]. However, large gaps remain in understanding the impact of elite sport during the preconception and pregnancy period as it relates to the menstrual cycle, fertility, pregnancy planning and necessary training modifications during pregnancy. The current studies measuring performance capabilities across the phases of the menstrual cycle are typically of low quality and only include eumenorrheic female athletes [72, 73].

A critical gap identified in this research pertained to the lack of pregnancy-specific nutritional advice and guidelines tailored for athletes, with many athletes receiving little to no guidance on nutritional considerations. This is of utmost concern as preconception and pregnancy health and nutrition influences short- and long-term maternal and child outcomes [8, 10, 12, 13]. In this study, team nutritionists often lacked this knowledge and directed athletes to the national maternity services where advice was perceived as generic and not tailored for athletes. Although athletes reinforced the desire found in the general population for nutritional information during pregnancy, such as what they can and can’t eat, they also highlighted the need for practical, athlete-specific recommendations [74]. Female athletes require nutritional considerations unique to their physiology and pregnancy status, especially as it relates to overall energy and macro- and micro-nutrient intakes [16, 75, 76]. Systematic review evidence indicates that female athletes have insufficient energy, carbohydrate, iron, and other key micronutrients when compared with health and sport recommendations [77]. Additionally, poor nutrition knowledge within female athlete populations contributes to suboptimal energy intakes [78, 79]. Addressing this gap necessitates the provision of evidence-based nutritional guidance specifically tailored to the unique requirements of females in elite sport.

However, this gap is compounded by the imbalance of female-specific sports science and medicine research, with females remaining significantly underrepresented in sport and exercise science studies [80]. Even in studies that include both sexes, the number of male participants often exceeds the total number of females. This disparity may partly stem from self-selection bias, where females are less willing or available to volunteer for certain investigations despite being eligible [81]. Additionally, the challenge of recruiting female athletes for research may reflect factors such as smaller team sizes, the historical lack of professional female athletes, and the limited availability of their time for participation [75]. Addressing these critical gaps requires a concerted effort from medical professionals, sporting organizations, and governing bodies to equip female athletes with tailored, evidence-based training and nutritional guidance that considers the unique physiological and performance demands of elite sport, especially during pregnancy. Furthermore, it needs proactive engagement from female athletes.

### Interpersonal

Access to support networks enable females to have both successful careers and raise children [82, 83]. There is a significant need to normalise conversations surrounding preconception health and pregnancy within the sporting community to help reduce the intrapersonal influences of fear, doubt and guilt expressed by the participants in this study. Many participants in this study planned their pregnancies around key competitions, which can often leave a narrow window to actively plan pregnancies and can even delay pregnancy intentions to later in life. This highlights the inherent challenges in reconciling athletic ambitions with family planning goals. Continuing to initiate dialogues and education pathways with teammates, coaching staff, other athletes who have experienced pregnancy, medical professionals and governing bodies can mitigate challenges associated with discussions around menstrual cycle and fertility and pregnancy planning and disclosure and foster a conducive environment to accessing specialised pregnancy resources related to physical and mental health support when needed [42].

Furthermore, in line with McGregor et al. [4], this study emphasises the significance of having other pregnant and postpartum elite athletes to connect with. Sharing experiences plays an important role in the culture shift of supporting and encouraging females to continue to participate in sport during pregnancy [84, 85]. A previous review and an England-based qualitative study showed that peer social support is instrumental in promoting new mothers’ well-being and mental health, as well as contributing to the building of confidence and self-esteem, and feelings of empowerment [86, 87]. These are traits often associated with being a mother, as well as being an athlete. UK Sport facilitation of informal (i.e. WhatsApp group, etc.) and formal (i.e. 2023 Pregnancy Guidelines) communication pathways and resources are an example of how support networks positively increase visibility of elite athlete mothers and champion their ability to navigate both elite sport and parenting.

### Intrapersonal

Becoming a mother is a transformative process, encompassing significant physical, psychological, social, athletic and financial changes [88]. The journey to become a mother intertwines with the demanding pursuit of elite athletic excellence, creating a complex intersection of physical, psychological and emotional adjustments [3]. Worryingly, despite positive progression in the field, this study's findings echo previous research that motherhood and an athletic career can still feel mutually exclusive [2, 5, 38, 39, 82]. Additionally, it continues to call attention to the challenges and decisions faced by athletes during, or when considering, pregnancy and their desire for greater support, understanding and opportunities to continue pursuing their athletic goals while starting a family [2, 5, 38, 39, 82]. Providing appropriate supports can help athletes navigate this transition [84].

In this study, participants continued to stress the difficulty they had in managing their identity as a pregnant elite athlete, specifically when navigating the necessary training and performance modifications. This struggle mirrors findings from previous studies, further illustrating how the two identities can both complement and conflict with each other across this transition from athlete to mother [85, 89, 90]. Wider evidence suggests that the two identities begin to complement each other as athletes return to sport postpartum [82, 85]. This period represents a pivotal juncture where identities converge and opportunities to promote holistic self-conceptualisation exist. Supporting athletes through this transition could enhance their ability to navigate the dual demands of pregnancy, motherhood, and high-performance sport [82, 85].

### Research and Policy Implications

Given the importance of health and wellbeing on maternal and child outcomes [28, 35], evidence-based research and policies in the UK and internationally must continue to evolve to better support elite athletes who train, compete, and participate in sports during their reproductive years and pregnancy. Initial research exploring an athlete's identity shift into motherhood to inform implementation of appropriate guidance, recommendations and structures which better support an athlete’s physical and mental health and wellbeing during pregnancy exists [4], however, it has yet to fully be translated to the athletes. There is a need to further understand what, when and how these various supports would best support athletes. High-quality research relating to hormones, menstruation, fertility, and maternal and foetal safety is needed to understand the impact of training on preconception and pregnancy health in elite athletes [16]. This would inform the necessary modifications to best support athletes during pregnancy. Further developing networks for athletes and disseminating preconception and pregnancy resources for those considering or experiencing pregnancy are essential to enable meaningful engagement with others who share similar experiences. These efforts will continue to highlight the importance of supporting pregnancy within the context of elite sport participation, ensuring athletes feel valued and supported during this period, while also increasing the visibility of those who have successfully navigated these challenges [35]. Additionally, while optimal performance may not be the primary outcome during pregnancy, further high-quality research is needed to best understand the nutrition modifications required in areas such as energy expenditure and macronutrient and micronutrient intake. This is necessary to support maternal and foetal health during pregnancy and postpartum and sufficiently support the athlete’s energy needs. Further, given the important role dietary supplementation plays in pregnancy and foetal health [8], there is a need to ensure robust testing of these supplements, as a matter of ethics, to alleviate female athlete’s concerns over job certainty. Lastly, the findings from this study are particularly relevant when considering that female elite athletes, who arguably have access to the most resources and support, still face significant challenges during pregnancy. This raises important questions about the experiences of sub-elite or recreational female athletes, who may have fewer resources and less institutional support. The implications of this research extend beyond elite sports and hold significance for females across all levels of physical activity, making it a timely and impactful contribution to ongoing discussions in behavioral nutrition and physical activity during preconception, pregnancy and postpartum.

### Strengths and Limitations

This qualitative study adds to the existing UK-based research in this field [2, 37] by exploring the pregnancy experiences of elite athletes from multiple sporting disciplines. Strengths of this study include using the 6-tiered Participant Classification Framework and including Tier 4: Elite/International and Tier 5: World Class athletes from a variety of team and individual sports [31]. All participants were either currently pregnant or pregnant within the last 4 years, reflecting recent experiences. Data quality was achieved by piloting interview questions with elite athletes, achieving information power, reflecting on positions and assumptions, and challenging assumptions through critical dialogue with qualitative research experts. However, limitations of this study need to be considered. Further in-depth or repeated interviews may have provided greater insights to take a more latent approach in the themes. While a more semantic approach was used in the analysis, it included elements of latent analysis [56], going beyond surface level meaning by providing further interpretation and aligning the themes to a SEF. Additionally, using a critical feminist approach in future research could identify historically male-centred infrastructures that could be impacting female health and provide further context. The lack of Paralympic athletes and athletes from ethnic minority groups limits the transferability of the research in elite sport, however, the purpose of qualitative sampling is to shed light on the particularities of a phenomena through multiple perspectives [48], which has been achieved here. This research focused only on elite athletes; however, many considerations may be relevant to sub-elite and recreational populations.

## Conclusion

This UK-based study highlights that despite existing supportive policies as well as increased research in the area, the same challenges and complexities faced by elite athletes during pregnancy and motherhood, as documented in previous research, remain. Interpreting the data through an SEF lens allowed participants’ experiences to be reflected across all levels of influence and helped us understand how macro- and micro-level influences affect athletes. This contextualisation has highlighted gaps in translation from policy to person, and advances discussions on the intersection of pregnancy, motherhood and sport participation. Key findings include the need for tailored pregnancy nutrition and supplementation guidance and support within the elite athlete population so that athletes do not risk their career to optimise pregnancy health. Additionally, it is essential to investigate methods to effectively translate policy guidance and practical application to this elite athlete population and their support teams, particularly as it relates to menstrual health, fertility, modified training behaviours during pregnancy and physical and mental health support. While this field is expanding in research and support for athletes, it is essential to reflect on the different socio-ecological levels that research is being conducted on, whether it is crossing levels, and how to effectively implement strategies that act across and is translated across these levels.

## Supplementary Information

Below is the link to the electronic supplementary material.

Supplementary file1 (PDF 201 KB)

Supplementary file2 (PDF 173 KB)

Supplementary file3 (PDF 115 KB)

## Declarations

### Availability of data and materials

The participants of this study did not give written consent for their data to be shared publicly, so due to the sensitive nature of the research supporting data is not available.
