# Supplementary material for: The Translation of Policy to Person: A Qualitative Analysis of Elite Athletes’ Perceptions of Pregnancy in the United Kingdom
Source: Sports Med. 2025 Mar 16;55(5):1293–306. doi: 10.1007/s40279-025-02191-9 (PMC12106561; doi:10.1007/s40279-025-02191-9)
Supplement: Supplementary file 2 — Supplementary file2 (PDF 173 KB) [file 40279_2025_2191_MOESM2_ESM.pdf]

Sports Medicine

The Translation of Policy to Person: A Qualitative Analysis of Elite Athletes Perceptions of Pregnancy in the United Kingdom

Catherine V. Caro[1,2], Storm Trow[1], Zoë Bell[1], Angela C. Flynn[2] and Fiona Lavelle[1*]
[1]*Department of Nutritional Sciences, School of Life Course & Population Sciences, King's College London, London, United Kingdom*
[2]*School of Population Health, Royal College of Surgeons in Ireland, Dublin, Ireland*

*Corresponding author; Dr Fiona Lavelle, King's College London, Department of Nutritional Sciences, 150 Stamford Street, London, SE1 9NH, United Kingdom; Fiona.lavelle@kcl.ac.uk

Supplementary Material 2. Semi-Structured Interview Topic guide.

1. Tell me about your involvement in sport, particularly at the elite level.
2. Tell me about your experience becoming pregnant while training or competing at the elite level?
3. Tell me about your experience with return to sport at the elite level following delivery?
4. What were the largest changes in training and nutrition behaviors during pregnancy and immediately after delivery?
5. What factors do you think support and/or limit female athletes' participation in sport?
6. What factors do you think limit elite athletes right to return to sport following delivery? What factors must be addressed in policies or regulations to support elite athletes who are pregnant?
7. What factors must be addressed in policies or regulations to support postpartum elite athletes and their return to sport?
8. What have we not discussed today, but you think is important to share with respect to the experiences of pregnant or postpartum elite athletes?
