# Supplementary material for: The Translation of Policy to Person: A Qualitative Analysis of Elite Athletes’ Perceptions of Pregnancy in the United Kingdom
Source: Sports Med. 2025 Mar 16;55(5):1293–306. doi: 10.1007/s40279-025-02191-9 (PMC12106561; doi:10.1007/s40279-025-02191-9)

Sports Medicine

The Translation of Policy to Person: A Qualitative Analysis of Elite Athletes Perceptions of Pregnancy in the United Kingdom

Catherine V. Caro[1,2], Storm Trow[1], Zoë Bell[1], Angela C. Flynn[2] and Fiona Lavelle[1*]
[1]*Department of Nutritional Sciences, School of Life Course & Population Sciences, King's College London, London, United Kingdom*
[2]*School of Population Health, Royal College of Surgeons in Ireland, Dublin, Ireland*

*Corresponding author; Dr Fiona Lavelle, King's College London, Department of Nutritional Sciences, 150 Stamford Street, London, SE1 9NH, United Kingdom; Fiona.lavelle@kcl.ac.uk

Supplementary Information 3. Theme Flowcharts

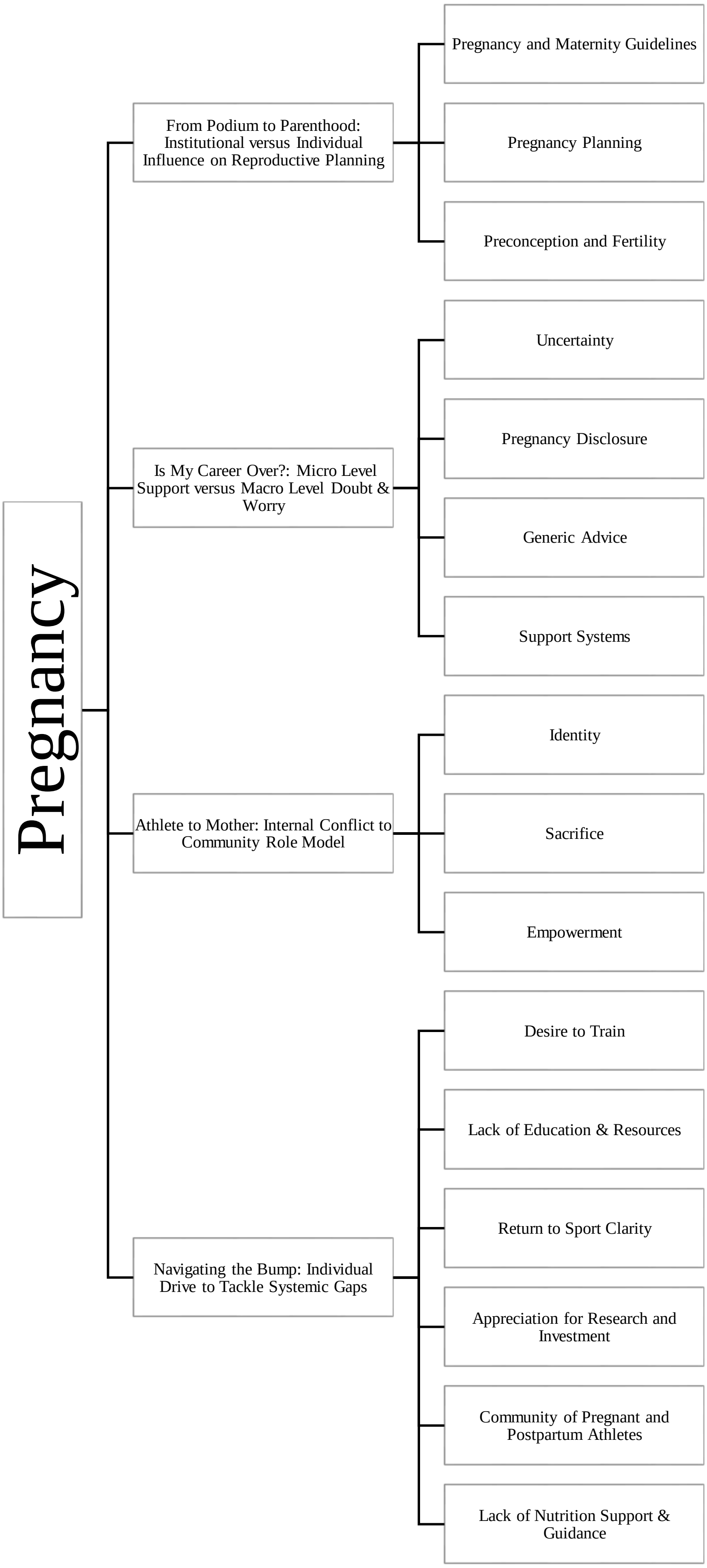

Supplement: Supplementary file 3 — Supplementary file3 (PDF 115 KB) [file 40279_2025_2191_MOESM3_ESM.pdf]
